# Drug-induced anaphylaxis in the emergency department: A prospective observational study

**DOI:** 10.14744/nci.2021.56667

**Published:** 2021-12-31

**Authors:** Fatma Sari Dogan, Vehbi Ozaydin

**Affiliations:** 1. Emergency Medicine Clinic, Fatih Sultan Mehmet Training and Research Hospital, Istanbul, Turkey; 2. Emergency Medicine Clinic, Medeniyet University, Goztepe Training and Research Hospital, Istanbul, Turkey

**Keywords:** Adrenaline, anaphylaxis, drug-induced anaphylaxis, emergency, Naranjo score

## Abstract

**Objective:**

Anaphylaxis is an acute, life-threatening, systemic hypersensitivity reaction. It is usually triggered by drugs, foods, and insect stings. The primary objective of our study is to determine the factors affecting drug-induced anaphylaxis to contribute to early diagnosis and treatment in these patients.

**Methods:**

Patients over 18 years old who were diagnosed drug-induced anaphylaxis in the Goztepe Hospital within a period of 1 year were evaluated prospectively. Patients demographical data, etiological factors, clinical findings, and treatment information were recorded.

**Results:**

Forty-four patients were enrolled in the study of which 25 (56.8%) were female. The median age of women and men was 54 (min: 22, max 82) and 44 (min 18, max 82), respectively. Twenty-three (52%) of them had a history of anaphylaxis. The most common causes of drug-induced anaphylaxis were antibiotics (36%) and nonsteroidal anti-inflammatory drugs (18%), respectively. Adrenaline was applied to 17 (38%) of the patients in the treatment.

**Conclusion:**

Antibiotics were the most common drugs causing drug-induced anaphylaxis and adrenaline was underused which is the first-line treatment in the anaphylaxis. Some clinicians refrain from administering adrenaline. The reasons underlying this approach should be investigated.

**A**naphylaxis is an acute, severe, life-threatening systemic hypersensitivity reaction caused by mediators secreted from mast cells and basophils. It is often provoked by drugs. Although tissue mast cells and circulating basophils play a major role in anaphylaxis, eosinophils, monocytes/macrophages, and endothelial cells also play a role. With the effect of chemical mediators in anaphylaxis, an increase in vascular permeability, systemic venous dilatation, a decrease in myocardial contractility, contraction in vessels, bronchioles, gastrointestinal tract, uterine smooth muscles, migration of eosinophils and neutrophils, increase in platelet aggregation, and degranulation are observed [1, 2]. Various signs and symptoms from urticaria to shock can be observed in the clinic. The diagnosis is made with clinical criteria [2–4]. Mortality can be prevented by early diagnosis and treatment. The primary drug in treatment is adrenaline [3].

The primary objective of our study is to determine the factors affecting drug-induced anaphylaxis (DIA) to contribute to early diagnosis and treatment in these patients.

## Materials and Methods

### Study Design and Data Collection

This is a prospective observational study. Patients over the age of 18 years who were diagnosed with DIA in the adult emergency department (ED) of Goztepe Training and Research Hospital within a period of 1 year were included in the study. Our hospital is a tertiary referral center and the number of the total population presented to the ED is approximately 180.000–200.000 in a year.

Patients who were diagnosed with anaphylaxis according to the clinical diagnostic criteria (Table 1) and who had a history of drug use before the complaint and who agreed to participate were included in the study. Patients under the age of eighteen and who did not consent to participate in the study were excluded from the study.

**Table 1.** Anaphylaxis diagnostic criteria

1.	If the allergen is unknown:
	• Skin and mucosa findings AND
	• Respiratory symptoms OR
	• Hypotension OR
	• Organ failure (hypotonia, syncope, collapse, incontinence)
2.	The presence of 2 or more after encountering a possible allergen:
	• Skin and mucosal findings (itching, rash, urticaria; tongue, soft palate, cheek, eyelid, or corneal swelling that may not be in 20% patients),
	• Respiratory symptoms (cough, wheezing, shortness of breath, change in voice)
	• Hypotension*, organ failure (syncope, collapse),
	• GIS symptoms (vomiting, cramp-like abdominal pain...)
3.	Rapidly developing hypotension after exposure to a known allergen

*Hypotension: Systolic blood pressure <90 mmHg or <30% of that person’s baseline value.

Patients’ age, gender, comorbid diseases, medications, the history of anaphylaxis, the drug that had caused anaphylaxis before, the route of drug administration (peroral, intravenous, intramuscular [IM], and dermal), clinical findings of anaphylaxis, presence of skin mucosa findings, gastrointestinal system (GIS) and respiratory symptoms, treatments for anaphylaxis, outcome of patients (discharge, hospitalization, and death), and Naranjo score [5] for anaphylaxis-drug relationship (Table 2) were recorded. The relationship between Naranjo score and DIA was defined as follows according to the score: If Naranjo score is >9 definite adverse effect (AE), if between 5 and 8 point is probable AE, if between 1 and 4 is possible AE, and if 0 is doubtful AE [5, 6].

**Table 2.** Naranjo score

		Yes	No	Unknown or not examined
1.	Are there clear reports that have previously reported this effect?	+1	0	0
2.	Has the adverse event developed after administration of the suspected drug?	+2	–1	0
3.	Has the adverse effect begun to improve when medication is released or a special antidote is applied?	+1	0	0
4.	Did the adverse effect repeat when the drug was applied again?	+2	–1	0
5.	Are there alternative reasons that may cause ımpact?	–1	+2	0
6.	Did the effect reappear when placebo was given?	–1	+1	0
7.	Is the drug detected at toxic concentrations in any body fluid?	+1	0	0
8.	Has the effect increased when the dose is increased, or is the effect decreased when it is decreased?	+1	0	0
9.	Is there a similar history of effect against a same or similar medication ın patient resume?	+1	0	0
10.	Has the adverse event been evidenced by any objective evidence?	+1	0	0

>9: Definite AE; 5–8: Probable AE; 1–4: Possible AE; 0: Doubtful AE.

### Statistical Analysis

Statistical Package for the Social Sciences, Inc., Chicago, IL, 17. 0 program was used for data analysis. Study data were evaluated in a 95% confidence interval and results were considered to be statistically significant if p<0.05. Chi-square test was used for categorical variables, frequency data were given as median, minimum, and maximum values. Shapiro–Wilk test was used in the analysis of normality between the gender groups in terms of age. Two groups suitable for normal distribution were compared with the Student’s t-test.

### Ethics Statement

The ethics approval was obtained from Hospital Scientific Review Board of GoztepeTraining and Research Hospital for this study (with no. 21/B-2012).

**Highlight key points**

• The most common drugs that caused DIA are antibiotics, nonsteroidal analgesics, paracetamol, and proton-pump inhibitor respectively.
• It is known that anaphylaxis should be remembered in patients with respiratory symptoms and hypotension however anaphylaxis should also be considered in critically ill patients, even if they have only gastrointestinal symptoms.
• Adrenaline has not been applied adequately although it is a first-line treatment.

## Results

A total of 44 patients (57% of female and 43% of male) were included in the study. The median age was 50 (min 18 and max 82), and the mean age was 50 (±16.5); the mean age of female and male (M) was 54 (±16) and 44 (±15) years, respectively. When the normality analysis was performed between the genders in terms of the mean age, it seemed that both groups fit the normal distribution (p=0.7 in F and p=0.7 in M) and there was no statistically significant difference between the two groups (p=0.058). Seventeen (40.9%) of the patients did not have any comorbid disease. Twenty-three (52%) of the patients had an anaphylaxis in their history. When the route of administration of the drug which is thought to cause anaphylaxis was examined; anaphylaxis developed with per-oral (PO) route in 35 (79.5%) of the patients, intravenous (IV) route (13.6%) in six of the patient, and IM (6.8%) route in three of the patients, respectively. Naranjo score was calculated as “probable” in 41 (93.2%) of patients and as “possible” in three (6.8%). Thirty-seven (84%) patients had skin, 30 (68%) had cardiovascular system (CVS), 17 (38%) had respiratory, and 5 (11%) had GIS symptoms. Clinical findings and managements are summarized in Tables 3–5.

**Table 3.** Clinical findings and managements

Variable	% (n=44)
Clinic findings	
Skin symptoms+Hypotension	47.7
Skin+Respiratory symptoms	18.2
Hypotension+Respiratory symptoms	11.4
Skin+Gastro-intestinal system symptoms	11.4
Hypotension+Skin+Respiratory symptoms	6.8
Hypotension	2.3
Respiratory symptoms	2.3
Management	
F+AH	6.8
F+AH+S+R	54.5
F+AH+S+R+A	**38.6**

*F: IV fluid resuscitation; AH: Antihistamines; A: Adrenaline; R: Ranitidine.

**Table 4.** Relationship between presence of clinical findings and adrenaline

Clinic findings	n (%)	n (adrenalin+)	p
Skin symptoms	37 (88.6)	12	0.08
Respiratory symptoms	17 (38.6)	16	**0.0001**
Cardiovascular symptoms	30 (68.2)	9	0.085
Gastrointestinal system symptoms	5 (11.4)	0	0.139

**Table 5.** Relationship between presence of clinical findings and steroid

Clinic findings	n (%)	n (Steroid+)	p
Skin symptoms	37 (84)	34	1.00
Respiratory symptoms	17 (38.6)	17	0.272
Cardiovascular symptoms	30 (68.2)	30	0.027
Gastrointestinal system symptoms	5 (11.4)	2	**0.001**

The most common drugs causing anaphylaxis were antibiotics (n=16, 36.4%) and nonsteroidal anti-inflammatory drugs (NSAIDs) (n=8, 18.2%), respectively (Table 6). Seven of the antibiotics that caused anaphylaxis were beta-lactam antibiotics (Three of them cephalosporin, three of them amoxicillin-clavulanate, and one of them procaine-penicillin), six were in quinolones (five of them moxifloxacin and one of them ciprofloxacin), and three were macrolides (two of them were spiramycin and one of them clarithromycin) groups.

**Table 6.** Drugs that cause anaphylaxis

Drugs	% (n=44)
Antibiotics	36.4
NSAID	18.2
Paracetamol	13.6
Proton-pump inhibitor	11.4
Multiple	9.1
Radiologic contrast	4.5
Unknown	6.8

NSAID: Nonsteroidal anti-inflammatory drug.

When the treatment for anaphylaxis is examined; it was observed that IV fluid resuscitation and antihistamines were applied to all patients and steroids were applied to 41 (93%) patients. The relationship between symptoms and steroid therapy was examined and significant relationship was found between the presence of GIS symptoms and steroid administration (p=0.001). Although adrenaline is the first-line treatment in anaphylaxis, adrenaline was applied to only 17 (38, 6%) patients. There was no patient using adrenaline autoinjector. Forty-three (97, 8%) of the patients were discharged from the ED. One patient referred to cardiology clinic because of chest pain and changed in electrocardiogram (ECG) after adrenaline administration. There were no deaths during the study.

One of the important results of our study, epinephrine was not used in all patients although it is the first choice in treatment by clinicians.

Therefore, the factors that might be associated with underuse of adrenaline by the clinicians were also investigated. There was no significant difference between genders in terms of injection of adrenaline in the treatment (p=0.3). Significant relationship was not found between with and without anaphylaxis history in terms of adrenaline administration (p=0.94). There was not significant relationship between drug route and adrenaline (p=0.82). There was a significant difference between the presence of bronchospasm and adrenaline administration in the clinic (p=0.0001). Clinically, there was no significant difference in terms of adrenaline treatment with GIS findings (p=0.13). Again, no statistically significant difference was observed between the presence or absence of hypotension, tachycardia, and adrenaline use in the clinic (p=0.08).

## Discussion

In this prospective study, we investigated the causes, clinical findings, and treatments of DIA. The diagnosis of DIA was made with clinical criteria of anaphylaxis and also made with the Naranjo score. It was observed that adrenaline has not been applied adequately although it is a first-line treatment.

Anaphylaxis can be defined as a potentially lethal, clinically diagnosed hypersensitivity reaction caused by suddenly developing mast cell and basophil mediator release (histamine, tryptase, chymase, and heparin) [1]. While urticaria is observed with the local effects of histamine, cardiovascular and hemodynamic changes are observed with systemic effects. Tryptase released from mast cells can also cause angioedema, hypotension, and increased tendency to clot [2].

The diagnosis is made with clinical criteria [7, 8]. Mediator levels can be measured for diagnosis anaphylaxis. The level of tryptase increases in 1–2 h, returns to normal in 5–6 h. Although histamine is the first released mediator, its normality does not exclude the diagnosis since its half-life is 20 min [7, 8]. In our study, the diagnosis was made by clinical criteria. In addition, Naranjo score was calculated for DIA. The criteria defined by Naranjo et al. [5] can be used to evaluate the relationship of anaphylaxis with the drug as an etiological factor. Naranjo criteria is a scoring system for predicting the likelihood of anaphylaxis being drug dependent and can be easily calculated. Naranjo score is calculated between 0 and 9 points according to the total value of the advers drug reaction and defined as “definitive,” “probable,” “possible,” “suspicious” [5, 6]. In our study, Naranjo score was “probable” in 93.2% and “possible” in 6.8% of our patients.

In the literature [8–11], the most common factors of DIA have been reported as antibiotics, NSAIDs, radiocontrast media, lidocaine, and ranitidine. Antibiotics [8–10] were the most common factors in some studies, while NSAIDs were the most common factors in others [12]. In our study, the most common factors were antibiotics, NSAIDs, paracetamol, and proton-pump inhibitor. Montañez et al. [8] reported that amoxicillin is the most common active agent in beta-lactam antibiotic anaphylaxis, and moxifloxacin is the most common active agent in non-beta-lactam antibiotic anaphylaxis. Similarly, in our study, the most common antibiotics were beta-lactams and second quinolones. When the anaphylaxis cases are analyzed in terms of average age, there are publications in the literature that examine in different age groups such as children (under 18), adult (18–65), and the elderly (>65 years) [9, 10, 12, 13]. Because the children were followed up in pediatric ward, they were not included in our study. The mean age of the patients was 50 in our study and it was similar to the average age of adults in the literature [9, 12, 13].

Anaphylaxis has been reported in the literature to be more serious with intravenous drug administration [14, 15]. Brown et al. [16] defined anaphylaxis as “serious” in the presence of symptoms such as hypoxia, consciousness alteration, hypotension, and incontinence, and reported 40% of reactions of oral drugs and 42% of injectable drugs as “serious”. In our study, anaphylaxis developed in 79.5% of the patients with PO drugs and 13.6% of the patients receiving IV drugs. Respiratory findings were observed in 50% of DIA with IV drugs and in 37% with PO drugs and we did not find a statistically significant relationship between respiratory distress and drug route.

Urticaria, angioedema, rhino-conjunctivitis, gastrointestinal symptoms, bronchospasm, and anaphylactic shock may appear in clinic of DIA [7, 8, 13]. In some studies, after skin findings, the most common symptoms were reported to be related with the respiratory system [8, 13]. However, in some other studies [10, 13], cardiovascular symptoms were recorded as the most frequently observed symptoms in DIA. In our study, 84% had skin symptoms, 68% had CVS, 38% had respiratory system, and 11% had GIS symptoms. Our results seemed to be similar with some publications in the literature [10, 13]. However, these results suggest that some cases of anaphylaxis may have been missed, especially if there are only GIS findings present in critical patients. Similarly, clinicians may be more alert for anaphylaxis when they see cases with respiratory symptoms and with hypotension. However, patients with comorbid respiratory diseases may also be overlooked as they have exacerbation of the disease process.

First-line treatment in anaphylaxis is IM adrenaline. If the agent is known, it should be removed, supportive oxygen and fluid should be started [1, 3, 8–11]. Considering the treatments applied in our study, it was seen that the most frequently applied treatment was hydration, antihistamines, and steroids. Significant relationship was found between the presence of GIS symptoms and steroid administration but this was not found clinically significant. We observed that the first-line treatment, adrenalin administration, remains in the background. There are different data on the use of adrenaline in the literature. While 8% of patients in Banerji et al. [9] study received adrenaline, adrenalin was used in 56% of DIA in the study of Kim et al. [10] Wang et al. [13] used it in 56% of DIA and Jares et al. [14] reported used in 27% of DIA. In our study, we found that adrenalin was administered to 37% of patients. Wang et al. [13] stated that most of the patients who received adrenaline were patients who developed respiratory symptoms, and similarly, in our study, we noted that adrenaline was applied more often to patients with respiratory symptoms, and this difference was statistically significant (p=0.0001).

Rarely, arrhythmia, angina, and myocardial infarction may occur due to the effect of adrenaline. Although the association of acute coronary events and anaphylaxis has been noted, the causal relationship between them is uncertain. Mast cells accumulate in areas of coronary atherosclerotic plaque, and mast cell degranulation can support plaque rupture during both acute myocardial events and anaphylaxis. In addition, the risk of myocardial ischemia may increase due to vasoconstriction in coronaries, and resulting in decreased intravascular volume, activation of coagulation pathways, and increased risk of myocardial ischemia due to endogenous or exogenous epinephrine [2]. In our study, one patient was referred to cardiology clinic because of developed chest pain and changed in ECG after adrenaline administration.

We thought that one of the possible reasons for not administering adrenaline is that clinicians might be afraid of cardiac side effects of adrenaline. Another reason may be that clinicians did not think that patients who present only with GIS symptoms may have a mortal outcome. In addition, the fact that mortality was not observed in any patient who did not receive adrenaline in our study group raises the question of whether it may be necessary to review the diagnostic criteria of anaphylaxis.

About 98% of our patients were followed up in the ED and discharged after treatment. In Banerji et al. [9] study, 71% of patients and in Jares et al. [14] study, 78% of them were discharged from the ED. Biphasic exacerbation may occur temporarily in anaphylaxis, therefore, follow-up time should be planned considering the second peak time of mediators, discharge should be done after this period.

### Limitations

Some cases of DIA may have been overlooked, especially those presenting with GIS symptoms. However, patients with comorbid respiratory disease may also be overlooked as they have exacerbation of the disease process.

### Conclusion

Anaphylaxis is a rapidly developing early hypersensitivity reaction that can be fatal if not diagnosed and treated. Drugs are the most common cause of anaphylaxis. In our study, we found that the most common drugs that caused anaphylaxis are antibiotics and nonsteroidal analgesics, respectively. Furthermore, adrenaline administration, which is the primary step in the treatment, lags behind. Although there are standard diagnostic criteria for anaphylaxis, some clinicians refrain from administering adrenaline. We think clinicians are hesitant about the possible side effects of adrenaline, but the reasons for this approach of clinicians should be investigated. Adrenaline should use in any doubtful anaphylaxis situation since overuse generally does not have any serious consequence for the patient, but not to use can result in increased severity and death.
